# Researches into the Physical History of Mankind

**Published:** 1841-10

**Authors:** 


Art. IX.—*Researches into the Physical History of Mankind.* By J. C. Prichard, m.d. f.r.s. &c. &c. Third Edition. Vol. III. Part I. —*London*, 1841. 8vo, pp. 507.

On former occasions (Br. and For. M. Rev., vol. III. 365, vol. V. 543,) we had the pleasure of noticing the first two volumes of this classical work, and of bestowing on them the humble meed of our praise. The cultivators of science throughout the world have, by their unanimous acclamation, justified and sanctioned that award. The present volume is worthy of its predecessors; and we know not that we could give it higher praise.

The portion of the work now entered on comprehends the ethnography or description, physical and moral, and history of all the different people who have inhabited Europe and Asia from the earliest period of which any record remains. The present volume is confined to the European tribes, and will be followed by a Second Part devoted to those of Asia. The whole, when completed, will be a lasting monument of the sagacity, judgment, industry, and learning of the author. We regret that the press of matter having a more immediate relation to medical science prevents our entering upon any detailed notice of this volume; but we can assure our readers that its perusal will afford to every scholar and, indeed, to every one interested (and who is not?) in the history and customs of his race, a treat of no ordinary description. And it will not please our readers and the readers of the present day only—it will please their successors for many generations. It is one of the few works that, in these later times, has been undertaken and executed in the spirit of former days, when men devoted their lives to the prosecution of one subject, and, thinking of the attainment of truth alone, were never troubled with the miserable jealousies, envyings, and fears which wither up every lofty enjoyment in the little minds who can never separate from the study of philosophy the consideration of paltry evanescent trifles having relation only to their own selfishness and vanity.

